# Attrition among Human Immunodeficiency Virus (HIV)- Infected Patients Initiating Antiretroviral Therapy in China, 2003–2010

**DOI:** 10.1371/journal.pone.0039414

**Published:** 2012-06-27

**Authors:** Hao Zhu, Sonia Napravnik, Joseph Eron, Stephen Cole, Ye Ma, David Wohl, Zhihui Dou, Yao Zhang, Zhongfu Liu, Decai Zhao, Myron Cohen, Fujie Zhang

**Affiliations:** 1 Department of Epidemiology, Gillings School of Global Public Health, University of North Carolina at Chapel Hill, North Carolina, United States of America; 2 Division of Infectious Diseases, School of Medicine, University of North Carolina at Chapel Hill, North Carolina, United States of America; 3 National Center for AIDS/STD Control and Prevention, Chinese Center for Disease Control and Prevention, Beijing, China; 4 Beijing Ditan Hospital Capital Medical University, Beijing, China; Indiana University, United States of America

## Abstract

**Background:**

Mortality and morbidity from HIV have dramatically decreased in both high- and low-income countries. However, some patients may not benefit from combination antiretroviral therapy (cART) because of inadequate access to HIV care, including attrition after care initiation.

**Methodology/Principal Findings:**

The study population included all HIV-infected patients receiving cART through the Chinese National Free Antiretroviral Treatment Program from 1 January 2003 to 31 December 2010 (n = 106,542). We evaluated retention in HIV care and used multivariable Cox proportional hazard models to identify independent factors predictive of attrition. The cumulative probability of attrition from cART initiation was 9% at 12 months, 13% at 18 months, 16% at 24 months and 24% at 60 months. A number of factors were associated with attrition, including younger age, male gender, and being single or divorced. Patients with higher CD4 cell counts at cART initiation were more likely to drop out of HIV care. The proportion of patients remaining in HIV care increased in more recent calendar years and among patients who initiated modern cART regimens.

**Conclusions/Significance:**

Retention in HIV care is essential for optimizing individual and public health outcomes. Attrition, even the degree observed in our study, can lead to premature morbidity and mortality, and possibly affect further transmission of HIV and HIV resistant drug variants. Effective strategies to promote retention in HIV care programs are needed. In China these strategies may include focusing particularly on younger male patients and those with higher CD4 cell counts at therapy initiation.

## Introduction

Local, national and international efforts have strived to make combination antiretroviral therapy (cART) available to patients with human immunodeficiency virus (HIV) around the globe, including China [1], [2], [3]. With these efforts and the increasing effectiveness of provided cART, mortality and morbidity from HIV have dramatically decreased in high- and low-income areas of the world [4], [5], [6]. However, some patients may not benefit from cART because of inadequate access to HIV care, including attrition after care initiation [7], [8], [9]. Estimates of attrition depend in large part on the definition of attrition used, the location and nature of the study population, and other study characteristics [10], [11], [12], [13].

Among HIV-infected patients initiating cART living in high-income areas of the world, attrition rates can be as low as 5% during the first year of treatment [5]. Studies conducted among patients living in low-income areas of the world have observed up to 50% attrition to HIV care, with many of these patients not returning to care because of death [14]. Better understanding of attrition among HIV-infected patients in care is essential to evaluating HIV clinical care provision and informing interventions for improving retention in care [15]. Retention in care could help improve individual clinical outcomes [16] and reduce risk of further HIV transmission in the community [17].

There were an estimated 740,000 HIV-infected patients living in China as of 2009 [18]. The major routes of HIV transmission in China include injection drug use (IDU), blood transfusion/former plasma donation, and heterosexual contact [19]. China initiated free cART provision through the National Free Antiretroviral Treatment Program (NFATP) in 2002. As of the end of 2009, over 80,000 HIV-infected patients had received cART through NFATP [4]. The effectiveness of this program in reducing mortality has been described previously [20], [21]. However, little is known about retention in HIV care among HIV-infected patients in China. The purpose of this study was to describe temporal trends in attrition and identify factors associated with greater risk of attrition among patients receiving cART through the Chinese NFATP Program from 2003 to 2010.

## Methods

### Study Population

The study population included all HIV-infected patients receiving cART through the Chinese NFATP from 1 January 2003 to 31 December 2010 (n = 106,542). The NFATP is managed by the Division of Treatment and Care in the National Center for AIDS/STD Control and Prevention, Chinese Center for Disease Control and Prevention. Detailed descriptions relevant to the management, process and methods of the Chinese NFATP have been published [22], [23]. The program used standardized paper based case report forms which were completed by local health workers and then faxed to a central office in the Division of Treatment and Care [24]. In 2010, a web-enabled electronic data collection system was implemented. At the visit when patients start cART, a standardized form for initial patient assessment is completed and baseline information is collected, including demographic data, suspected HIV infection exposure route, clinical symptoms and signs, and laboratory test results. Subsequent follow up visits are scheduled for 2, 4, 8 and 12 weeks following cART initiation, and then every 3 months thereafter. Patients who transfer to another NFATP clinical site retain the same patient identification number that is assigned at cART initiation and therefore patients can be tracked centrally across NFATP clinical sites. Information on death, including reason and date of death, was available through the NFATP treatment withdrawal forms. These forms were also completed by local health workers and sent to central NFATP offices.

From its inception, NFATP provided free cART to all HIV-infected patients with a CD4 cell counts below 200 cells/µL, a total lymphocyte counts <1200 cells/µL or a World Health Organization (WHO) stage III or IV. At the beginning of 2008, patients with CD4 cell counts <350 cells/µL became eligible for free cART in accordance with updated WHO guidelines [24].

For these analyses we included all HIV-infected adults who were ART-naïve at the time they initiated free cART through NFATP. Henan Province did not submit data to NFATP until July 1, 2006; therefore, patients residing in Henan Province who initiated cART before that date were excluded from this analysis (n = 16,609) [25]. Additionally we excluded patients who did not have any recorded follow-up (n = 2,896), consistent with prior work in this area [26], and those who initiated cART less than 210 days (approximately seven months) prior to 31 December 2010 to allow for adequate follow-up (n = 14,195).

The data were analyzed anonymously. This study was approved by the Institutional Review Board at the University of North Carolina at Chapel Hill. Individual informed consent was not needed because this analysis used currently existing data collected during the course of routine treatment and care.

### Measurements

Attrition was defined as not having a visit for at least 210 days. This is approximately seven months, which allows leniency for those who miss a standard three-month NFATP visit but are seen within four weeks of the following scheduled NFATP visit. Moreover, this seven month definition is roughly similar to the definition of six months used in prior studies [27], [28]. In sensitivity analyses, we assessed the effect of using different time intervals to define attrition, including 180, 270 and 360 days from last observation. Factors measured at baseline included age, gender, marital status, suspected HIV exposure route, initial cART regimen, CD4 cell counts, alanine aminotransferase and hemoglobin at cART initiation. Baseline symptoms recorded include fever, cough, sputum production, dyspnea, chest pain, night sweat, diarrhea, nausea, projectile vomiting, headache, declining vision, blurred vision, rash, thrush, oral hairy leukoplakia, and lymphadenopathy. For these analyses, we summed the number of symptoms reported for each patient as a measure of clinical symptom severity. Antiretroviral therapy regimens were categorized as: neviripine (NVP) with lamivudine (3TC) and either zidovudine (AZT) or stavudine (D4T); efavirenz (EFV) with 3TC and either AZT or D4T; NVP and didanosine (DDI) and either AZT or D4T; and all other regimens. Health care setting (general hospital, infectious diseases hospital, centers for diseases control clinic, health care center at township level, village clinic and prison hospital), area of residence (eastern, central and western region) and calendar year of cART initiation were also included.

### Statistical Analysis

Person-time was calculated as time from cART initiation to the first of attrition or date of censoring. Patients were censored at the date of death or 31 December 2010, whichever occurred first. The complement of the Kaplan-Meier survival curve was used to plot the cumulative incidence of attrition by levels of factors of interest. The log-rank test statistic was used to assess differences in survival curves.

Cox proportional hazard models were used to estimate unadjusted and adjusted hazard ratios and 95% confidence intervals (CI), and we assessed for deviations from the proportional hazard assumption. Based on the bivariable results, we identified factors predictive of the outcome with a P value<0.2. These factors were jointly entered into a model. Then backward elimination was used to sequentially remove covariates with the highest P-values such that the final model included only factors predictive of the outcome defined as a P value<0.05.

Given the relationship between type of antiretroviral therapy available through NFATP and calendar year of starting cART, we explored the relationship between type of therapy and attrition in models stratified by calendar year of cART initiation. As a sensitivity analysis we administratively censored follow-up time at 12 and 24 months following cART initiation rather than at 31 December 2010. We also performed sensitivity analyses to assess whether results were robust to excluding patients with no follow-up. Data were analyzed using SAS version 9.2 (SAS Institute, Cary NC, USA). All hypothesis testing was 2-sided, with α level of 0.05.

## Results

Between 1 January 2003 and 31 May 2010, 67,732 HIV-infected patients who initiated cART through the Chinese NFATP were included in this analysis. Over one-third (37%) of patients were women, 51% were infected through heterosexual contact, 23% through blood transfusion/former plasma donation, and 22% through IDU (Table S1). At cART initiation, the median age was 38 years (interquartile range [IQR]: 32–45), and the median CD4 cell counts was 131 cells/µL (IQR: 42–220). The median year of cART initiation was 2008 (IQR: 2007–2009). The initial cART regimen prescribed was predominantly NVP and 3TC with either AZT or D4T (70%).

**Figure 1:**
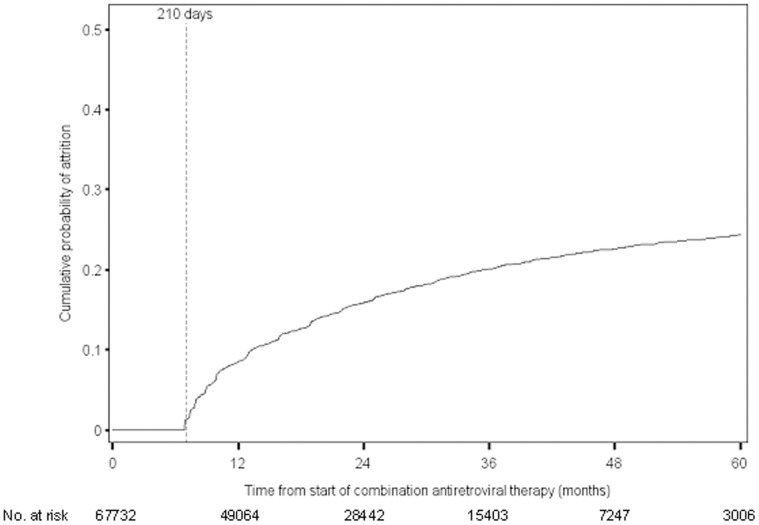
Cumulative probability of attrition among HIV-infected patients who initiated combination antiretroviral therapy in the China National Free Antiretroviral Treatment Program, 2003–2010.

Patients contributed a median of 20 months of follow-up (IQR: 11–34) and 8 visits (IQR: 5–13) from cART initiation to first attrition event, death or censoring. The overall person-time contributed was 137,792 person-years. Of the 67,732 HIV-infected patients, 9,969 were lost to HIV care, and the remaining 57,763 were administratively censored (7,390 of these 57,763 were censored at death). Among those administratively censored the median time on study was 22 months (IQR: 13–37), and among those who died the median time to death was 4 months (IQR: 1–11). The cumulative probability of attrition from cART initiation was 9% at 12 months, 13% at 18 months, 16% at 24 months and 24% at 60 months (Figure 1).

**Figure 2:**
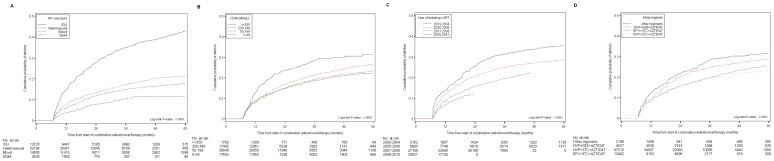
Cumulative probability of attrition among HIV-infected patients who initiated combination antiretroviral therapy in the China National Free Antiretroviral Treatment Program, 2003–2010; (A) by HIV exposure group; (B) by CD4 cell counts; (C) by calendar year of therapy initiation; and (D) by type of initial regimen. Note: IDU  = injection drug use; MSM = men who have sex with men; blood  =  blood transfusion/former plasma donation; NVP = nevirapine; 3TC = lamivudine; AZT = zidovudine; D4T = stavudine; DDI = didanosine; EFV = efavirenz.

A number of factors were associated with attrition in unadjusted and adjusted analyses, including younger age, male gender and being single or divorced (Table S2). Patients infected through IDU were almost twice as likely to be lost to HIV care as those infected by heterosexual contact [adjusted Hazard Ratio (aHR) 1.87, 95% CI: 1.76, 1.98] (Table S2, Figure 2A). Patients initiating cART in the central region of China were less likely to be lost to HIV care than patients living in other areas of China, and patients who received HIV care at larger and more centralized health care settings were more likely to be lost to care than patients receiving care at village level clinics. In multivariable analyses the direction of these relationships persisted although some were notably attenuated, particularly the association between attrition and type of health care setting.

Patients with higher CD4 cell counts at cART initiation were more likely to be lost to care (Figure 2B). The hazard of attrition among patients with CD4 cell counts greater than 350 cells/µL was 1.6 times greater than for patients with CD4 cell counts less than 50 cells/µL (HR = 1.57; 95% CI:1.41, 1.74). The hazard of attrition was relatively similar among patients with CD4 cell counts less than 350 cells/mL at cART initiation. The relationship between CD4 cell count and attrition persisted after multivariable adjustment. For patients with CD4 cell counts greater than 350 cells/µL, the aHR was 1.76 (95% CI: 1.55, 2.00) (Table S2). Patients with lower hemoglobin (HR = 1.24; 95% CI: 1.12, 1.37) and higher ALT (HR = 1.28; 95% CI: 1.16, 1.41) were also more likely to be lost, although ALT did not remain predictive in multivariable analyses. Patients with more than four symptoms at cART initiation had a higher hazard of attrition, even after accounting for other characteristics.

Notably, attrition decreased with increasing cART initiation calendar year (Figure 2C) and among patients who initiated modern cART regimens (including 3TC with either NVP or EFV) (Figure 2D). These relationships were consistent in multivariable adjusted analyses (Table S2). To further assess the relationship between calendar year of cART initiation and type of regimen received, we compared cART regimens containing and not containing lamivudine. We observed a persistent effect of lamivudine associated with less attrition in models adjusting for cART initiation calendar year and those stratified by cART initiation calendar year (Table 1).

**Table 1:** Association between initial combination antiretroviral regimen (Lamivudine/Non-lamivudine) and time to attrition overall and stratified by calendar year of therapy initiation.

	HR (95% CI)
**Unadjusted**	
Non-lamivudine	1.33 (1.25–1.41)
Lamivudine	1.00
**Adjusted all factors but NOT calendar year†**	
Non-lamivudine	1.81 (1.65–1.98)
Lamivudine	1.00
**Adjusted all factors and calendar year‡**	
Non-lamivudine	1.39 (1.26–1.54)
Lamivudine	1.00
**Adjusted stratified by calendar year of cART initiation** *	
2003–2004	
Non-lamivudine	N/A**
Lamivudine	N/A**
2005–2006	
Non-lamivudine	1.44 (1.24–1.67)
Lamivudine	1.00
2007–2008	
Non-lamivudine	1.81 (1.55–2.11)
Lamivudine	1.00
2009–2010	
Non-lamivudine	1.41 (0.97–2.07)
Lamivudine	1.00

**Note:** HR = hazard ratio; 95% CI = 95% Confidence Interval; **†** Includes initial regimen (lamivudine/Non-lamivudine) and patients’ age, gender, marriage status, HIV exposure category, CD4 cell counts, hemoglobin level, clinical symptoms, area of residence, health care setting but excludes the year of starting therapy. ‡ Includes all factors plus the year of starting therapy.

* Includes all factors but stratifies by the year of starting therapy.

** Because lamivudine was only available after 2005 in China; therefore we did not make estimates during 2003–2004.

We assessed the effect of cART initiation calendar year with varying lengths of follow-up. We fit models to data where we administratively censored follow-up at 12 and at 24 months (Table 2). The reductions in attrition in more recent calendar years persisted in all models and were comparable to results observed relying on all patient observation time available. In additional sensitivity analyses, using different time intervals to define attrition, including 180, 270 and 360 days from last observation, our results were consistent irrespective of the definition of attrition we employed (data not shown). Finally we evaluated the effect of excluding patients who were only seen once by NFATP at their initial patient visit (n = 2,896). Our results were unaltered when we included this group of patients in our main analyses (data not shown).

**Table 2:** Association between the calendar year of combination antiretroviral therapy initiation and time to attrition stratified by observation time.

	HR (95% CI)
**Follow-up time truncated at 12 months of follow-up:**	
**Unadjusted**	
2003–2004	2.18 (1.92–2.48)
2005–2006	1.81 (1.68–1.96)
2007–2008	1.30 (1.22–1.39)
2009–2010	1.00
**Adjusted all factors but NOT initial regimens†**	
2003–2004	4.28 (3.57–5.14)
2005–2006	1.77 (1.61–1.93)
2007–2008	1.20 (1.12–1.29)
2009–2010	1.00
**Adjusted all factors and initial regimens‡**	
2003–2004	4.08 (3.35–4.96)
2005–2006	1.85 (1.68–2.03)
2007–2008	1.23 (1.14–1.32)
2009–2010	1.00
**Follow-up time truncated at 24 months of follow-up:**	
**Unadjusted**	
2003–2004	2.23 (2.03–2.46)
2005–2006	1.74 (1.64–1.86)
2007–2008	1.29 (1.23–1.37)
2009–2010	1.00
**Adjusted all factors but NOT initial regimens†**	
2003–2004	3.58 (3.09–4.16)
2005–2006	1.72 (1.60–1.85)
2007–2008	1.22 (1.15–1.30)
2009–2010	1.00
**Adjusted all factors and initial regimens‡**	
2003–2004	3.14 (2.67–3.69)
2005–2006	1.77 (1.64–1.91)
2007–2008	1.25 (1.17–1.32)
2009–2010	1.00

**Note:** HR = hazard ratio; 95% CI = 95% Confidence Interval; **†** Includes the year of starting cART and patients’ age, gender, marriage status, HIV exposure category, CD4 cell counts at enrollment, hemoglobin level, counts of baseline symptoms, area of residence, health care setting and initial regimens but excludes initial regimens. ‡ Includes all factors plus initial regimens.

## Discussion

At one year following cART initiation the cumulative probability of attrition was 9% and rose to 24% at 5 years among patients receiving free antiretroviral therapy in China between 2003 and 2010 through the NFATP. This degree of retention in care is slightly better than that observed in low-income areas of the world (approximately 15% at one year) and slightly worse than that observed in high-income areas of the world (approximately 5% at one year) [5]. In a recent study conducted in Uganda attrition rates of 16%, 30% and 39% were observed at 1, 2 and 3 years following cART initiation, respectively [27].

Attrition was associated with both individual and contextual factors. Consistent with prior studies patients who acquired HIV through IDU were at substantial risk of being lost to care. In this study 22% of patients accessing HIV care were IDU, in comparison to 39% of HIV infections reported nationally [19]. This observation highlights that patients with IDU encounter problems with both initiating and remaining in HIV care. In contrast to previous studies where either CD4 cell counts did not affect attrition or patients with lower CD4 cell counts had poorer retention [7], [29], among Chinese patients in NFATP we observed greater attrition among patients with higher CD4 cell counts. This result suggests that unrecognized mortality may not be a substantial underlying cause of attrition in this study population. However, attrition was higher among patients with lower hemoglobin, which has been associated with greater mortality [2], [26].

We observed encouraging results that in more recent calendar years and with the provision of newer cART regimens patients were retained in care longer. In multivariable analyses we did not observe substantial differences in attrition by type of clinic. Other studies conducted in low-income settings have found a similar attrition equality across large and small health care settings [30], [31], [32].

Our study is one of the largest to evaluate retention in HIV care and the only study to date to be carried out in China. However there are limitations to the present study. We were not able to link the study population with death registries, nor was there active follow-up of patients lost to care. Therefore, our estimate of attrition includes patients who died but their death was not reported to the clinic where they were receiving HIV care. In prior studies conducted primarily in Africa as many as one-half of patients not returning to HIV care were found to be deceased when active tracing was available [33], [34], [35]. We do not know the outcomes of patients lost to follow-up in NFATP; however, given the relatively high CD4 cell counts among patients lost to care it is possible that mortality may not have been a primary cause of attrition from HIV care in this population. We were also unable to evaluate the effect of longitudinal CD4 cell counts or HIV RNA levels on retention as these data were not available in the early years of the NFATP program. As additional information on these biomarkers becomes available it will be important to evaluate how they are associated with retention in HIV care. Additionally, given the unique nature of the Chinese NFATP program our results may not generalize to clinics in other areas of the world. For example, as part of NFATP all patients received free antiretroviral therapy as well as other needed HIV care. In other settings it has been shown that retention increases in programs that offer free ART [36]. We excluded patients with only one visit from our primary analyses; however, including these patients did not affect our results. We also treated deaths as a censoring event to be consistent with prior literature [7]. It is possible that not treating death as a competing risk may have resulted in some bias. Finally, we were not able to take into account hepatitis B (HBV) co-infection among HIV patients in this study. Although we were able to assess the effect of ALT on retention this marker alone may not have captured the entire effect of HBV co-infection, particularly given the high prevalence of HBV in China [37] and its relationship to greater mortality among HIV-infected patients [38].

These results have important implications for the management of HIV in China. Treatment of HIV, even at higher CD4 cell counts, clearly reduces transmission [17], and earlier treatment of HIV has measurable improvement on patient outcomes [39]. But these benefits can only be achieved if HIV infection is detected, patients are efficiently referred for care, and following cART initiation patients remain in active clinical follow-up. Any substantial attrition, and certainly the level of attrition documented in this study, can erode the benefits of antiviral therapy and give rise to increased transmission of HIV and HIV drug resistance. Reassuringly to date prevalence of transmitted HIV drug resistance remains relatively low in China [40]. Our results identify patients most likely to be lost, and several factors that help to explain loss. These results must be used to design programs to keep HIV-infected people in care, and over a very long period of time.

## Supporting Information

Table S1 **Note: ALT = alanine aminotransferase; cART = Combination Antiretroviral Therapy; NVP =  nevirapine; 3TC = lamivudine; AZT =  zidovudine; D4T = stavudine; DDI = didanosine; EFV = efavirenz.**
(DOC)

Table S2 *Adjusted hazard ratios are based on one model including all characteristics listed in column except ALT. Note: HR  =  hazard ratio; 95% CI  = 95% Confidence Interval; ALT = alanine aminotransferase; cART = Combination Antiretroviral Therapy; NVP = nevirapine; 3TC = lamivudine; AZT = zidovudine; D4T = stavudine; DDI = didanosine; EFV = efavirenz.
(DOC)

## References

[1] Gilks CF, Crowley S, Ekpini R, Gove S, Perriens J (2006). The WHO public-health approach to antiretroviral treatment against HIV in resource-limited settings.. Lancet.

[2] May M, Boulle A, Phiri S, Messou E, Myer L (2010). Prognosis of patients with HIV-1 infection starting antiretroviral therapy in sub-Saharan Africa: a collaborative analysis of scale-up programmes.. Lancet.

[3] Zhang F, Dou Z, Ma Y, Zhang Y, Zhao Y (2010). Effect of earlier initiation of antiretroviral treatment and increased treatment coverage on HIV-related mortality in China: a national observational cohort study.. Lancet Infect Dis.

[4] Zhang F, Dou Z, Ma Y, Zhao Y, Liu Z (2009). Five-year outcomes of the China National Free Antiretroviral Treatment Program.. Ann Intern Med 151: 241–251, W-252.

[5] Braitstein P, Brinkhof MW, Dabis F, Schechter M, Boulle A (2006). Mortality of HIV-1-infected patients in the first year of antiretroviral therapy: comparison between low-income and high-income countries.. Lancet.

[6] ART Cohort Collaboration (2008). Life expectancy of individuals on combination antiretroviral therapy in high-income countries: a collaborative analysis of 14 cohort studies.. Lancet.

[7] Mocroft A, Kirk O, Aldins P, Chies A, Blaxhult A (2008). Loss to follow-up in an international, multicentre observational study.. HIV Med.

[8] Maskew M, MacPhail P, Menezes C, Rubel D (2007). Lost to follow up: contributing factors and challenges in South African patients on antiretroviral therapy.. S Afr Med J.

[9] Brinkhof MW, Dabis F, Myer L, Bangsberg DR, Boulle A (2008). Early loss of HIV-infected patients on potent antiretroviral therapy programmes in lower-income countries.. Bull World Health Organ.

[10] MacPherson P, Moshabela M, Martinson N, Pronyk P (2009). Mortality and loss to follow-up among HAART initiators in rural South Africa.. Trans R Soc Trop Med Hyg.

[11] Chasombat S, McConnell MS, Siangphoe U, Yuktanont P, Jirawattanapisal T (2009). National expansion of antiretroviral treatment in Thailand, 2000–2007: program scale-up and patient outcomes.. J Acquir Immune Defic Syndr.

[12] Karcher H, Omondi A, Odera J, Kunz A, Harms G (2007). Risk factors for treatment denial and loss to follow-up in an antiretroviral treatment cohort in Kenya.. Trop Med Int Health.

[13] Lanoy E, Mary-Krause M, Tattevin P, Dray-Spira R, Duvivier C (2006). Predictors identified for losses to follow-up among HIV-seropositive patients.. J Clin Epidemiol.

[14] Brinkhof MW, Pujades-Rodriguez M, Egger M (2009). Mortality of patients lost to follow-up in antiretroviral treatment programmes in resource-limited settings: systematic review and meta-analysis.. PLoS One.

[15] Mugavero MJ, Norton WE, Saag MS (2011). Health care system and policy factors influencing engagement in HIV medical care: piecing together the fragments of a fractured health care delivery system.. Clin Infect Dis.

[16] Mugavero MJ, Lin HY, Willig JH, Westfall AO, Ulett KB (2009). Missed visits and mortality among patients establishing initial outpatient HIV treatment.. Clin Infect Dis.

[17] Cohen MS, Chen YQ, McCauley M, Gamble T, Hosseinipour MC (2011). Prevention of HIV-1 infection with early antiretroviral therapy.. N Engl J Med.

[18] UNAIDS (2010). Report on the Global AIDS Epidemic Joint United Nations Programme on HIV/AIDS, Geneva..

[19] (2007). State Council AIDS Working Committee Office, UN Theme Group on HIV/AIDS in the People’s Republic of China.. A Joint Assessment of HIV/AIDS Prevention, Treatment, and Care in China: Beijing.

[20] Zhang F, Dou Z, Yu L, Xu J, Jiao JH (2008). The effect of highly active antiretroviral therapy on mortality among HIV-infected former plasma donors in China.. Clin Infect Dis.

[21] Dou Z, Chen RY, Wang Z, Ji G, Peng G (2010). HIV-infected former plasma donors in rural Central China: from infection to survival outcomes, 1985–2008.. PLoS One.

[22] Ma Y, Zhang F, Zhao Y, Zang C, Zhao D (2009). Cohort profile: the Chinese national free antiretroviral treatment cohort.. Int J Epidemiol.

[23] Zhang FJ, Pan J, Yu L, Wen Y, Zhao Y (2005). Current progress of China’s free ART program.. Cell Res.

[24] Zhang F (2008). China Free Antiretroviral Therapy Manual, 2008 Edition..

[25] Dou Z, Chen RY, Xu J, Ma Y, Jiao JH (2010). Changing baseline characteristics among patients in the China National Free Antiretroviral Treatment Program, 2002–09.. Int J Epidemiol.

[26] Ekouevi DK, Balestre E, Ba-Gomis FO, Eholie SP, Maiga M (2010). Low retention of HIV-infected patients on antiretroviral therapy in 11 clinical centres in West Africa.. Trop Med Int Health.

[27] Geng EH, Bangsberg DR, Musinguzi N, Emenyonu N, Bwana MB (2010). Understanding reasons for and outcomes of patients lost to follow-up in antiretroviral therapy programs in Africa through a sampling-based approach.. J Acquir Immune Defic Syndr.

[28] Geng EH, Emenyonu N, Bwana MB, Glidden DV, Martin JN (2008). Sampling-based approach to determining outcomes of patients lost to follow-up in antiretroviral therapy scale-up programs in Africa.. Jama.

[29] Lawn SD, Myer L, Harling G, Orrell C, Bekker LG (2006). Determinants of mortality and nondeath losses from an antiretroviral treatment service in South Africa: implications for program evaluation.. Clin Infect Dis.

[30] Geng EH, Nash D, Kambugu A, Zhang Y, Braitstein P (2010). Retention in care among HIV-infected patients in resource-limited settings: emerging insights and new directions.. Curr HIV/AIDS Rep.

[31] Chan AK, Mateyu G, Jahn A, Schouten E, Arora P (2010). Outcome assessment of decentralization of antiretroviral therapy provision in a rural district of Malawi using an integrated primary care model.. Trop Med Int Health.

[32] Bedelu M, Ford N, Hilderbrand K, Reuter H (2007). Implementing antiretroviral therapy in rural communities: the Lusikisiki model of decentralized HIV/AIDS care.. J Infect Dis.

[33] Yu JK, Chen SC, Wang KY, Chang CS, Makombe SD (2007). True outcomes for patients on antiretroviral therapy who are “lost to follow-up” in Malawi.. Bull World Health Organ.

[34] Bisson GP, Gaolathe T, Gross R, Rollins C, Bellamy S (2008). Overestimates of survival after HAART: implications for global scale-up efforts.. PLoS One.

[35] Dalal RP, Macphail C, Mqhayi M, Wing J, Feldman C (2008). Characteristics and outcomes of adult patients lost to follow-up at an antiretroviral treatment clinic in johannesburg, South Africa.. J Acquir Immune Defic Syndr.

[36] Zachariah R, Van Engelgem I, Massaquoi M, Kocholla L, Manzi M (2008). Payment for antiretroviral drugs is associated with a higher rate of patients lost to follow-up than those offered free-of-charge therapy in Nairobi, Kenya.. Trans R Soc Trop Med Hyg.

[37] Liu J, Fan D (2007). Hepatitis B in China.. Lancet.

[38] Matthews G (2007). The management of HIV and hepatitis B coinfection.. Curr Opin Infect Dis.

[39] Kitahata MM, Gange SJ, Abraham AG, Merriman B, Saag MS (2009). Effect of early versus deferred antiretroviral therapy for HIV on survival.. N Engl J Med.

[40] Liao L, Xing H, Shang H, Li J, Zhong P (2010). The prevalence of transmitted antiretroviral drug resistance in treatment-naive HIV-infected individuals in China.. J Acquir Immune Defic Syndr.

