# Supplementary material for: Attrition among Human Immunodeficiency Virus (HIV)- Infected Patients Initiating Antiretroviral Therapy in China, 2003–2010
Source: PLoS One. 2012 Jun 27;7(6):e39414. doi: 10.1371/journal.pone.0039414 (PMC3384674; doi:10.1371/journal.pone.0039414)
Supplement: Table S1 — Note: ALT = alanine aminotransferase; cART = Combination Antiretroviral Therapy; NVP = nevirapine; 3TC = lamivudine; AZT = zidovudine; D4T = stavudine; DDI = didanosine; EFV = efavirenz. (DOC) [file pone.0039414.s001.doc]

**Table S1. Characteristics of 67,732 HIV-infected patients at combination antiretroviral therapy initiation, the China National Free Antiretroviral Treatment Program 2003 - 2010**

| **Characteristic** | N (%) or Median (IQR) |
| --- | --- |
| Age (years) | |
| Median (IQR) | 38 (32 - 45) |
| 18-29 | 11509 (17%) |
| 30-44 | 38200 (56%) |
| ≥45 | 18023 (27%) |
| Gender | |
| Female | 24930 (37%) |
| Male | 42798 (63%) |
| Marital status |  |
| Single | 10875 (16%) |
| Married | 46434 (69%) |
| Divorced | 4864 (7%) |
| Widowed | 5399 (8%) |
| HIV exposure | |
| Blood transfusion/former plasma donation | 14555 (23%) |
| Intravenous drug use | 13735 (22%) |
| Homosexual transmission | 2826 (4%) |
| Heterosexual transmission | 32194 (51%) |
| Area of residence | |
| Eastern region | 8359 (12%) |
| Central region | 21278 (32%) |
| Western region | 38095 (56%) |
| Health care setting | |
| General hospital | 26801 (40%) |
| Infectious diseases hospital | 12321 (18%) |
| Centers for diseases control clinic | 16019 (24%) |
| Health care center at township level | 9496 (14%) |
| Village clinic | 2000 (3%) |
| Prison hospital | 646 (1%) |
| CD4 cell counts (cells/ μL) | |
| Median (IQR) | 131 (42 - 220) |
| 0-49 | 17692 (28%) |
| 50-199 | 26648 (42%) |
| 200-349 | 17465 (27%) |
| ≥350 | 1760 (3%) |
| Hemoglobin (g/L) | |
| Median (IQR) | 124 (108 - 140) |
| 0-79 | 2612 (4%) |
| ≥80 | 59269 (96%) |
| ALT (U/L) | |
| Median (IQR) | 28 (18 - 44) |
| ≥100 | 2421 (4%) |
| 0-99 | 58875 (96%) |
| Number of baseline symptoms | |
| ≥4 | 18308 (27%) |
| 2-3 | 15257 (22%) |
| 1 | 9214 (14%) |
| 0 | 24953 (37%) |
| Initiation cART regimen | |
| NVP+3TC+AZT | 22179 (33%) |
| NVP+3TC+D4T | 25336 (37%) |
| NVP+DDI+AZT | 2557 (4%) |
| NVP+DDI+D4T | 1970 (3%) |
| EFV+3TC+AZT | 7052 (10%) |
| EFV+3TC+D4T | 6440 (10%) |
| Other regimens | 2198 (3%) |
| Year of cART initiation | |
| 2003-2004 | 2182 (3%) |
| 2005-2006 | 9880 (15%) |
| 2007-2008 | 27149 (40%) |
| 2009-2010 | 28521 (42%) |
