# Supplementary material for: Attrition among Human Immunodeficiency Virus (HIV)- Infected Patients Initiating Antiretroviral Therapy in China, 2003–2010
Source: PLoS One. 2012 Jun 27;7(6):e39414. doi: 10.1371/journal.pone.0039414 (PMC3384674; doi:10.1371/journal.pone.0039414)
Supplement: Table S2 — *Adjusted hazard ratios are based on one model including all characteristics listed in column except ALT. Note: HR = hazard ratio; 95% CI = 95% Confidence Interval; ALT = alanine aminotransferase; cART = Combination Antiretroviral Therapy; NVP = nevirapine; 3TC = lamivudine; AZT = zidovudine; D4T = stavudine; DDI = didanosine; EFV = efavirenz. (DOC) [file pone.0039414.s002.doc]

**Table S2. Clinical and demographic characteristics associated with attrition among HIV-infected patients at combination antiretroviral therapy initiation, the China National Free Antiretroviral Treatment Program** 2003 - 2010

| **Characteristic** | **Unadjusted HR (95% CI)** | ***Adjusted HR (95% CI)** |
| --- | --- | --- |
| Age (years) | | |
| 18-29 | 1.52 (1.43 -1.61) | 1.10 (1.02 - 1.19) |
| 30-44 | 1.28 (1.22 -1.34) | 0.94 (0.88 - 1.00) |
| ≥45 | 1 | 1 |
| Gender | | |
| Male | 1.30 (1.24 - 1.35) | 1.09 (1.03 - 1.15) |
| Female | 1 | 1 |
| Marital status | | |
| Divorced | 1.41 (1.31 - 1.51) | 1.30 (1.20 - 1.41) |
| Single | 1.62 (1.55 - 1.70) | 1.26 (1.18 - 1.34) |
| Widowed | 0.95 (0.88 - 1.03) | 1.10 (1.01 - 1.21) |
| Married | 1 | 1 |
| HIV exposure | | |
| Blood transfusion/former plasma donation | 0.83 (0.78 - 0.88) | 0.74 (0.67 - 0.82) |
| Intravenous drug use | 2.20 (2.10 - 2.30) | 1.87 (1.76 - 1.98) |
| Homosexual transmission | 0.53 (0.45 - 0.62) | 0.52 (0.43 - 0.61) |
| Heterosexual transmission | 1 | 1 |
| Area of residence | | |
| Eastern region | 0.92 (0.86 - 0.97) | 1.04 (0.96 - 1.12) |
| Central region | 0.61 (0.58 - 0.64) | 0.67 (0.61 - 0.74) |
| Western region | 1 | 1 |
| Health care setting | | |
| General hospital | 2.60 (2.22 - 3.05) | 1.19 (0.96 - 1.48) |
| Infectious diseases hospital | 2.09 (1.77 - 2.46) | 1.09 (0.87 - 1.36) |
| Centers for diseases control clinic | 1.67 (1.42 - 1.96) | 1.02 (0.82 - 1.27) |
| Health care center at township level | 1.78 (1.50 - 2.10) | 1.42 (1.15 - 1.76) |
| Prison hospital | 5.99 (4.89 - 7.33) | 2.33 (1.80 - 3.01) |
| Village clinic | 1 | 1 |
| CD4 cell counts (cells/ μL) | | |
| ≥350 | 1.57 (1.41 - 1.74) | 1.76 (1.55 - 2.00) |
| 200-349 | 1.12 (1.06 - 1.19) | 1.07 (1.00 - 1.14) |
| 50-199 | 1.01 (0.96 - 1.06) | 0.99 (0.93 - 1.05) |
| 0-49 | 1 | 1 |
| Hemoglobin (g/L) | | |
| 0-79 | 1.24 (1.12 - 1.37) | 1.24 (1.10 - 1.39) |
| ≥80 | 1 | 1 |
| ALT (U/L) |  |  |
| ≥100 | 1.28 (1.16 - 1.41) |  |
| 0-99 | 1 |  |
| Number of baseline symptom | | |
| ≥4 | 1.07 (1.02 - 1.13) | 1.07 (1.01 - 1.14) |
| 2-3 | 0.95 (0.90 - 1.00) | 0.98 (0.92 - 1.04) |
| 1 | 0.96 (0.90 - 1.02) | 0.96 (0.89 - 1.04) |
| 0 | 1 | 1 |
| Initiation cART regimen | | |
| NVP+3TC+AZT | 0.90 (0.83 - 0.97) | 0.95 (0.88 - 1.03) |
| NVP+3TC+D4T | 0.92 (0.85 - 0.99) | 0.86 (0.79 - 0.93) |
| NVP+DDI+AZT | 1.19 (1.07 - 1.33) | 1.58 (1.35 - 1.86) |
| NVP+DDI+D4T | 1.18 (1.06 - 1.32) | 0.92 (0.77 - 1.10) |
| EFV+3TC+AZT | 1 | 1 |
| EFV+3TC+D4T | 0.89 (0.81 - 0.98) | 0.83 (0.75 - 0.92) |
| Other regimens | 1.32 (1.18 - 1.47) | 1.29 (1.12 - 1.48) |
| Year of cART initiation | | |
| 2003-2004 | 2.24 (2.05 - 2.44) | 3.29 (2.84 - 3.81) |
| 2005-2006 | 1.71 (1.61 - 1.82) | 1.70 (1.58 - 1.83) |
| 2007-2008 | 1.31 (1.24 - 1.38) | 1.25 (1.17 - 1.32) |
| 2009-2010 | 1 | 1 |
